# Supplementary material for: Acquisitive plants exhibit stronger phenological shifts in response to warming: insights from meta-analysis and long-term monitoring
Source: Nat Commun. 2026 Mar 26;17:4430. doi: 10.1038/s41467-026-70474-4 (PMC13183894; doi:10.1038/s41467-026-70474-4)
Supplement: Supplementary file 1 — Supplementary Information [file 41467_2026_70474_MOESM1_ESM.pdf]

Supplementary information

Acquisitive plants exhibit stronger phenological shifts in response to warming: insights from meta-analysis and long-term monitoring

Content:

Supplementary Tables 1-10

Supplementary Figs. 1-17

Supplementary Table 1. Correlations between the first principal component (PC1) of the economic spectrum and five foliar traits in Figure 2a, Figure 3a and Figure 3d. Statistical significance ($P < 0.05$) was tested using two-sided tests without adjustments for multiple comparisons. $P < 0.05$ suggests significant correlations and highlighted in **bold**. *r*, the correlation coefficient between factors; FN, foliar nitrogen concentration; FP, foliar phosphorus concentration; SLA, specific leaf area; LT, leaf thickness; FDMC, foliar dry-matter concentration; PC1, the first principal component of the economic spectrum.

Data source		*r* or *P*	FN	FP	SLA	LT	FDMC
Meta-analysis data (Figure 2a)	PC1	*r*	0.680	0.568	0.620	-0.634	-0.610
		P	**0**	**0**	**0**	**0**	**0**
long-term ground data (Figure 3a)	PC1	*r*	0.649	0.564	0.905	-0.738	-0.631
		P	**0**	**0**	**0**	**0**	**0**
long-term ground data (Figure 3d)	PC1	*r*	0.634	0.561	0.798	-0.182	-0.639
		P	**0**	**0**	**0**	$\mathbf{8.790e^{-9}}$	**0**

Supplementary Table 2. Weighted average effect size of different phenological parameters. Statistical significance (P < 0.05) was tested using two-sided tests from multi-level meta-analytic linear mixed-effects models without adjustments for multiple comparisons. P < 0.05 suggests the significant influences and highlighted in **bold**. CI, confidence interval (d per °C).

Foliar phenology	Category	Estimate (95% CI)	*P*-value
Spring	Overall	-1.42 (-2.03, -0.80)	**$6.513e^{-6}$**
	Conservative	-1.93 (-3.57, -0.28)	**0.022**
	Intermediate	-0.96 (-1.90, -0.02)	**0.044**
	Acquisitive	-2.66 (-3.47, -1.84)	**$1.610e^{-10}$**
	Deciduous	-1.40 (-2.08, -0.71)	**$6.913e^{-5}$**
	Evergreen	-0.78 (-1.82, 0.25)	0.137
	Angiosperms	-1.27 (-1.98, -0.57)	**0.0004**
	Gymnosperms	-2.11 (-3.33, -0.88)	**0.001**
Autumnal	Overall	1.38 (0.68, 2.09)	**0.0001**
	Conservative	-2.46 (-6.71, 1.78)	0.256
	Intermediate	1.18 (0.43, 1.93)	**0.002**
	Acquisitive	1.87 (0.10, 3.63)	**0.038**
	Deciduous	1.53 (0.79, 2.27)	**$4.889e^{-5}$**
	Evergreen	0.97 (-0.19, 2.13)	0.100
	Angiosperms	1.67 (1.01, 2.34)	**$8.374e^{-7}$**
	Gymnosperms	-1.89 (-4.80, 1.01)	0.202

Supplementary Table 3. Phylogenetic signals explaining effects of warming on foliar phenology temperature sensitivity. Values of Blomberg's *K* and *P* represent explaining power, phylogenetic signal explains variances in warming effects when $P < 0.05$. Statistical significance ($P < 0.05$) was tested using two-sided tests without adjustments for multiple comparisons.

Foliar phenology sensitivity	Category	K	*P*
Spring	Overall	0.037	0.177
	woody	0.036	0.250
	herbaceous	0.051	0.524
Autumnal	Overall	0.046	0.297
	woody	0.035	0.500
	herbaceous	0.074	0.325

Supplementary Table 4. Weighted average effect sizes of different phenological parameters after excluding phylogenetic relatedness. Statistical significance ($P < 0.05$) was tested using two-sided tests from multi-level meta-analytic linear mixed-effects models without adjustments for multiple comparisons. $P < 0.05$ suggests the significant influences and highlighted in **bold**. CI, confidence interval (d per °C).

Foliar phenology	Category	Estimate (95% CI)	*P*-value
Spring	Overall	-1.37 (-3.33, 0.59)	0.172
	Conservative	-1.87 (-3.81, 0.07)	0.059
	Intermediate	-1.46 (-4.11, 1.19)	0.280
	Acquisitive	-2.55 (-3.70, -1.41)	**$1.234e^{-5}$**
Autumnal	Overall	0.11 (-1.70, 1.91)	0.909
	Conservative	-2.73 (-7.41, 1.95)	0.253
	Intermediate	0.96 (-0.33, 2.25)	0.144
	Acquisitive	2.08 (-0.47, 4.62)	0.110

Supplementary Table 5. Future predictions of foliar phenological changes based on or without considering plant traits by 2100 under different future climate change scenarios. The shifts in foliar phenology are expressed as mean ± standard deviation. SSP, Shared Socioeconomic Paths.

Prediction methods	Climate scenarios	Shifts in foliar phenology (d)	
		Spring	Autumnal
Traits integration projections	SSP1-2.6	-0.94±1.04	3.18±2.64
	SSP5-8.5	-1.84±1.78	6.22±4.37
Climate-only projections	SSP1-2.6	-4.94±2.29	4.91±2.28
	SSP5-8.5	-9.74±3.31	9.67±3.28

Supplementary Table 6. Predictor variables of effects of warming effects on foliar phenology temperature sensitivity in meta-analysis.

Predictor variables	Variable description	Abbreviation	Value range (Unit)
Environmental conditions	Latitude		-36.86~79.12 (°)
	Longitude		-156.78~147.28 (°)
	Mean annual temperature	MAT	0.2~10.0 (℃)
	Mean annual precipitation	MAP	99~2500 (mm)
	Ecosystem type	Ecotype	Grassland/forest/tundra/shrubland/farmland/desert
Experimental conditions	Warming magnitude		0.2~15 (℃)
	Warming duration		1~15 (year)
	Warming method		Cable/chamber/heater
Plant traits	Foliar nitrogen concentration	FN	9.11~39.82 (mg/g)
	Foliar phosphorus concentration	FP	0.65~5.45 (mg/g)
	Foliar nitrogen/phosphorus ratio	FN:FP	5.16~30.59 (g/g)
	Leaf area	LA	15.00~21129.30 (mm^2)
	Specific leaf area	SLA	2.50~54.69 (mm^2/mg)
	Foliar carbon concentration	FC	348.92~519.84 (mg/g)
	Foliar carbon/nitrogen ratio	FC:FN	10.90~53.07 (g/g)
	Foliar dry-matter concentration	FDMC	0.14~0.58 (g/g)
	Leaf thickness	LT	0.07~0.86 (mm)
	Leaf lifespan	LLS	2.37~82.99 (month)
	Plant life form		Woody/herbaceous

Supplementary Table 7. List of plant phenophases description and corresponding classification in USA National Phenology Network.

Phenophases description	Phenological classification
All leaf buds broken (lilac/honeysuckle)	Spring phenology
Breaking leaf buds (lilac/honeysuckle)	Spring phenology
Leaves (forbs)	Spring phenology
Initial growth (forbs)	Spring phenology
Increasing leaf size	Spring phenology
Leaves	Spring phenology
Breaking leaf buds	Spring phenology
>=75% of full leaf size (deciduous)	Spring phenology
First leaf (historic lilac/honeysuckle)	Spring phenology
Full leaf (historic lilac/honeysuckle)	Spring phenology
Emerging leaves (1 location)	Spring phenology
Young unfolded leaves (evergreen, 1 location)	Spring phenology
Young leaves (tree/shrub)	Spring phenology
Leaves (grasses)	Spring phenology
Initial growth (grasses/sedges)	Spring phenology
Young leaves (forbs)	Spring phenology
Leaves (sedges)	Spring phenology
All leaves colored (deciduous)	Autumnal phenology
>=50% of leaves fallen (deciduous)	Autumnal phenology
>=50% of leaves colored (deciduous)	Autumnal phenology
All leaves fallen (deciduous)	Autumnal phenology
Colored leaves	Autumnal phenology
All leaves withered (herbaceous and grasses)	Autumnal phenology
Falling leaves	Autumnal phenology

Supplementary Table 8. Egger's regression test and Rosenberg fail-safe numbers for publication bias. Egger's regression test was used to identify publication bias; $P > 0.05$ suggests the absence of the publication bias. If there is evidence of publication bias, the Rosenthal's fail-safe number was utilized to determine if the potential impact of unpublished studies could alter our findings. Rosenberg fail-safe numbers greater than 5N+10 (N, the number of observations) indicates that the results were not affected by unpublished articles. Statistical significance ($P < 0.05$) was tested using two-sided tests without adjustments for multiple comparisons.

Foliar phenology	Category	*z* value for Egger's regression test	*P* value for Egger's regression test	Rosenberg failsafe number	5N+10
Spring	Overall	5.99	$2.038e^{-9}$	5576289	9465
	Conservative	-2.44	0.015	83551	1230
	Intermediate	6.16	$7.139e^{-10}$	574447	4275
	Acquisitive	-0.53	0.596		
	Deciduous	5.32	$1.023e^{-7}$	3101889	6375
	Evergreen	2.70	0.007	109884	2675
	Angiosperms	6.16	$7.381e^{-10}$	3129466	7690
	Gymnosperms	-2.15	0.031	102112	1360
Autumnal	Overall	4.04	$5.380e^{-5}$	1463749	5545
	Conservative	-3.40	0.001	5376	790
	Intermediate	3.01	0.003	473847	2545
	Acquisitive	-0.66	0.507		
	Deciduous	1.75	0.081		
	Evergreen	4.96	$7.014e^{-7}$	2408	1475
	Angiosperms	2.90	0.004	1398554	4375
	Gymnosperms	1.49	0.136		

Supplementary Table 9. Interactive effects of foliar functional traits and experimental and environmental factors on foliar phenology temperature sensitivity of warming. When analyzing the interactions between traits and MAT, MAP, and ecosystem types, only field experiments data is included in the analysis. Statistical significance ($P < 0.05$) was tested using two-sided tests without adjustments for multiple comparisons. $P < 0.05$ suggests the significant influences and highlighted in **bold**. FN, foliar nitrogen concentration; FP, foliar phosphorus concentration; FN:FP, foliar nitrogen/phosphorus ratio; LA, leaf area; SLA, specific leaf area; FC: foliar carbon concentration; FC:FN, foliar carbon/nitrogen ratio; FDMC, foliar dry-matter concentration; LT, leaf thickness; LLS, leaf lifespan; MAT, mean annual temperature; MAP, mean annual precipitation; Ecotype, ecosystem type.

Interaction effect	Spring phenology sensitivity		Autumnal phenology sensitivity	
	Q_M	P	Q_M	P
FN*Warming method	10.0718	**0.018**	68.3188	**9.777e^{-15}**
FN*Warming duration	9.5329	**0.002**	3.8662	**0.049**
FN*Warming magnitude	0.8526	0.356	0.5752	0.448
FN*MAT	4.4828	**0.034**	9.3510	**0.002**
FN*MAP	12.1163	**0.001**	45.2658	**1.720e^{-11}**
FN*Ecotype	19.7251	**0.001**	64.2988	**1.566e^{-12}**
FP*Warming method	7.7246	0.052	24.2520	**2.213e^{-5}**
FP*Warming duration	3.3919	0.066	0.1501	0.698
FP*Warming magnitude	1.4880	0.223	0.3465	0.556
FP*MAT	7.5339	**0.006**	1.9034	0.168
FP*MAP	9.3681	**0.002**	17.5002	**2.873e^{-5}**
FP*Ecotype	25.8129	**9.701e^{-5}**	29.4099	**1.927e^{-5}**
FN:FP*Warming method	2.8343	0.418	30.2918	**1.198e^{-6}**
FN: FP*Warming duration	22.1060	**2.580e^{-6}**	0.0988	0.753
FN:FP*Warming magnitude	0.0019	0.965	0.2927	0.589
FN:FP*MAT	0.5099	0.475	4.8451	**0.028**
FN:FP*MAP	3.8741	**0.049**	14.9497	**0.0001**
FN:FP*Ecotype	3.0325	0.695	20.5800	**0.0001**
LA*Warming method	5.7910	0.122	9.3284	**0.025**
LA*Warming duration	0.3961	0.529	3.2084	0.073
LA*Warming magnitude	2.9729	0.085	1.2460	0.264
LA*MAT	4.3767	**0.036**	3.0369	0.081
LA*MAP	4.3316	**0.037**	7.3324	**0.007**
LA*Ecotype	6.0108	0.198	17.4595	**0.001**
SLA*Warming method	2.7756	0.428	62.9033	**1.408e^{-13}**
SLA*Warming duration	6.6354	**0.010**	14.7443	**0.0001**
SLA*Warming magnitude	0.6414	0.423	6.3791	**0.012**
SLA*MAT	0.3644	0.546	15.3565	**8.901e^{-5}**
SLA*MAP	1.3409	0.247	44.0764	**3.158e^{-11}**
SLA*Ecotype	6.1693	0.290	54.5657	**1.603e^{-10}**
FC*Warming method	2.4399	0.486	33.0129	**3.201e^{-7}**
FC*Warming duration	25.5023	**4.419e^{-7}**	8.6659	**0.003**

Interaction effect	Spring phenology sensitivity		Autumnal phenology sensitivity	
	Q_M	*P*	Q_M	*P*
FC*Warming magnitude	0.0214	0.884	12.5213	**0.0004**
FC*MAT	0.7367	0.391	0.4398	0.507
FC*MAP	5.2549	**0.022**	0.2661	0.606
FC*Ecotype	8.0718	0.152	37.5628	**$4.619e^{-7}$**
FC:FN*Warming method	8.6436	**0.034**	79.8534	**$3.300e^{-17}$**
FC:FN*Warming duration	25.6890	**$4.011e^{-7}$**	45.6946	**$1.382e^{-11}$**
FC:FN*Warming magnitude	2.3968	0.122	52.3090	**$4.742e^{-13}$**
FC:FN*MAT	0.2680	0.605	13.7965	**0.0002**
FC:FN*MAP	0.4905	0.484	52.5206	**$4.258e^{-13}$**
FC:FN*Ecotype	11.0192	0.051	73.8896	**$1.586e^{-14}$**
FDMC*Warming method	4.3449	0.227	64.8258	**$5.465e^{-14}$**
FDMC*Warming duration	25.8130	**$3.762e^{-7}$**	33.2395	**$8.148e^{-9}$**
FDMC*Warming magnitude	1.1923	0.275	29.4857	**$5.633e^{-8}$**
FDMC*MAT	0.3427	0.558	0.9433	0.331
FDMC*MAP	1.5543	0.213	24.3649	**$7.971e^{-7}$**
FDMC*Ecotype	5.8163	0.325	55.9369	**$2.067e^{-11}$**
LT*Warming method	3.2815	0.350	71.9116	**$1.663e^{-15}$**
LT*Warming duration	10.3699	**0.001**	73.0231	**$1.281e^{-17}$**
LT*Warming magnitude	1.2225	0.269	61.0985	**$5.429e^{-15}$**
LT*MAT	0.0017	0.967	58.1594	**$2.417e^{-14}$**
LT*MAP	1.0945	0.296	68.7896	**$1.095e^{-16}$**
LT*Ecotype	9.5164	**0.049**	74.2932	**$1.306e^{-14}$**
LLS*Warming method	1.4976	0.683	93.0921	**$4.746e^{-20}$**
LLS*Warming duration	4.7354	**0.030**	91.9553	**$8.866e^{-22}$**
LLS*Warming magnitude	2.5469	0.111	84.6927	**$3.485e^{-20}$**
LLS*MAT	0.4228	0.516	76.5000	**$2.202e^{-18}$**
LLS*MAP	0.3402	0.560	85.6207	**$2.180e^{-20}$**
LLS*Ecotype	4.9587	0.421	96.8606	**$7.352e^{-21}$**

Supplementary Table 10. Correlations between spring or autumnal phenology temperature sensitivity and environmental and experimental factors in meta-analysis data and two long-term ground phenological observation networks data sets. For meta-analysis data, only field experiments data is included in the analysis. Statistical significance (P < 0.05) was tested using two-sided tests without adjustments for multiple comparisons. $P < 0.05$ suggests significant correlations and highlighted in **bold**. MAT, mean annual temperature; MAP, mean annual precipitation. *, $P < 0.05$; **, $0.001 < P < 0.01$; ***, $P < 0.001$.

Data source	Foliar phenology	*r* or *P*	MAT	MAP	Latitude	Warming duration	Warming magnitude
Meta-analysis data	Spring	*r*	-0.187	-0.030	-0.196	0.174	-0.031
		P	**$3.582e^{-5}$**	**$5.058e^{-1}$**	**$1.550e^{-5}$**	**$1.228e^{-4}$**	**$4.942e^{-1}$**
	Autumnal	*r*	-0.024	0.054	-0.089	-0.151	-0.202
		P	0.673	0.345	0.122	**0.008**	**0.0004**
long-term ground data	Spring	*r*	0.154	-0.025	-0.196		
		P	**0.005**	0.654	**0.0004**		
	Autumnal	*r*	0.102	0.095	-0.100		
		P	0.087	0.109	0.094		

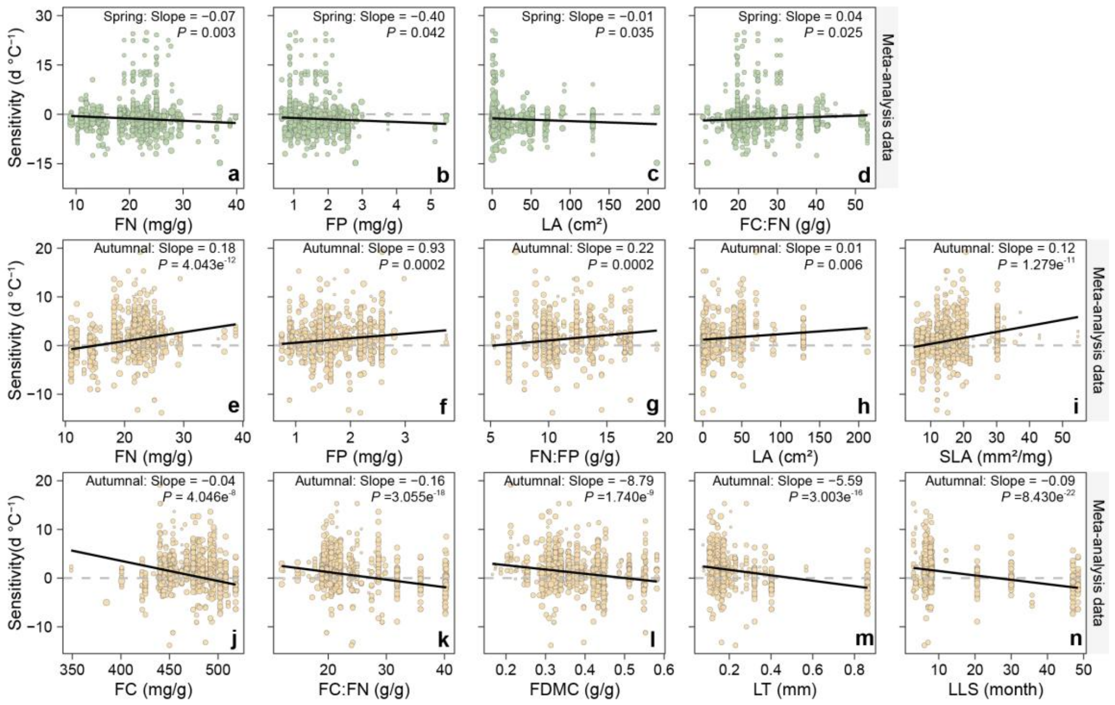

Supplementary Fig.1 Relationships between foliar functional traits and foliar phenology temperature sensitivity based on experimental manipulations. The foliar phenology includes spring phenology (**a**-**d**) and autumnal phenology (**e**-**n**). Statistical significance ($P < 0.05$) was tested using two-sided tests from multi-level meta-analytic linear mixed-effects models without adjustments for multiple comparisons. Regression lines are shown when $P < 0.05$. The size of the points is proportional to the weight in this meta-analysis. FN, foliar nitrogen concentration; FP, foliar phosphorus concentration; FN:FP, foliar nitrogen/phosphorus ratio; LA, leaf area; SLA, specific leaf area; FC: foliar carbon concentration; FC:FN, foliar carbon/nitrogen ratio; FDMC, foliar dry-matter concentration; LT, leaf thickness; LLS, leaf lifespan. Source data are provided as a Source Data file.

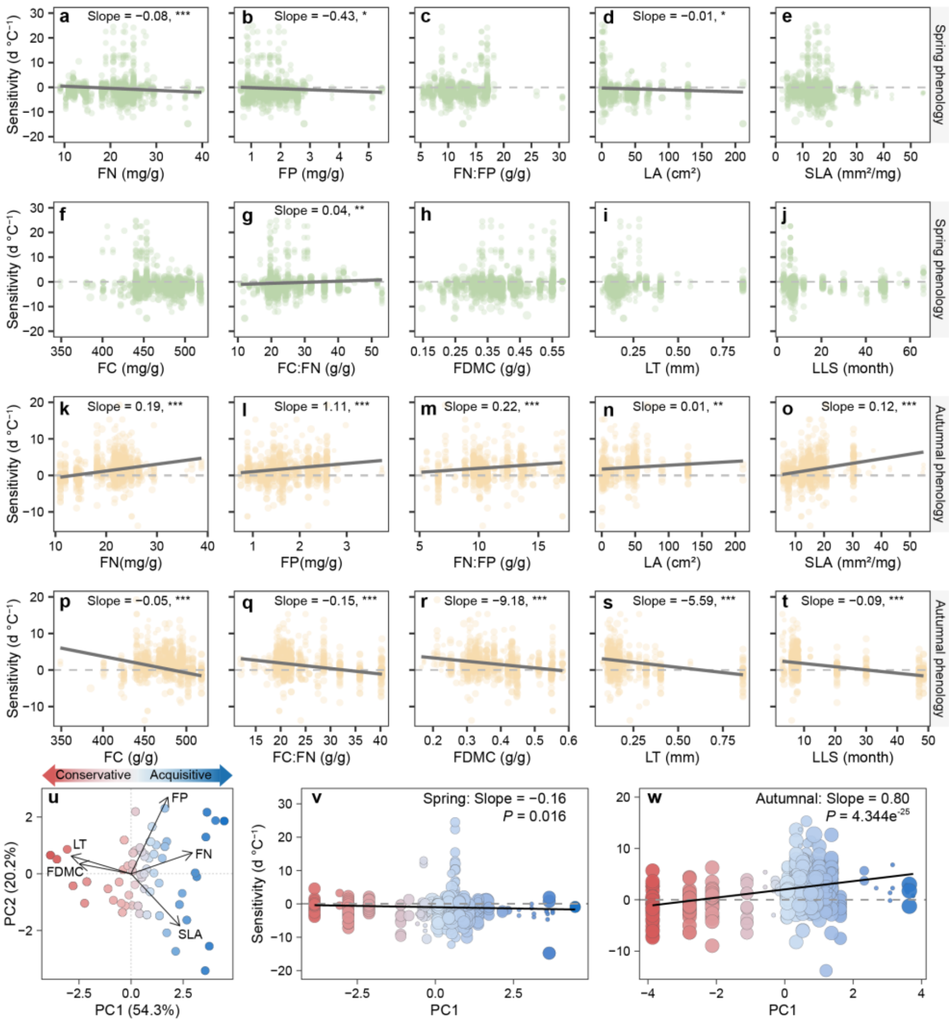

Supplementary Fig.2 Relationship between foliar functional traits and foliar phenology temperature sensitivity based on experimental manipulations after excluding environmental and experimental factors. Only field experiments data is included in the analysis. The foliar phenology includes spring phenology (**a**–**j**) and autumnal phenology (**k**–**t**). **u**, The leaf economic spectrum based on five plant traits, where plants with redder and bluer colors are more resource-conservative and -acquisitive, respectively, in their resource use. **v**, **w**, Relationships between the first principal component (PC1) of the economic spectrum and the sensitivities of spring and autumnal phenology to temperature after excluding environmental and experimental factors. Statistical significance (P < 0.05) was tested using two-sided tests from multi-level meta-analytic linear mixed-effects models without adjustments for multiple comparisons. Regression lines are shown when P < 0.05. The size of the points is proportional to the weight in this meta-analysis. FN, foliar nitrogen concentration; FP, foliar phosphorus concentration; FN:FP, foliar nitrogen/phosphorus ratio; LA, leaf area; SLA, specific leaf area; FC: foliar carbon concentration; FC:FN, foliar carbon/nitrogen ratio; FDMC, foliar dry-matter concentration; LT, leaf thickness; LLS, leaf lifespan. *, P < 0.05; **, 0.001 < P < 0.01; ***, P < 0.001. Source data are provided as a Source Data file.

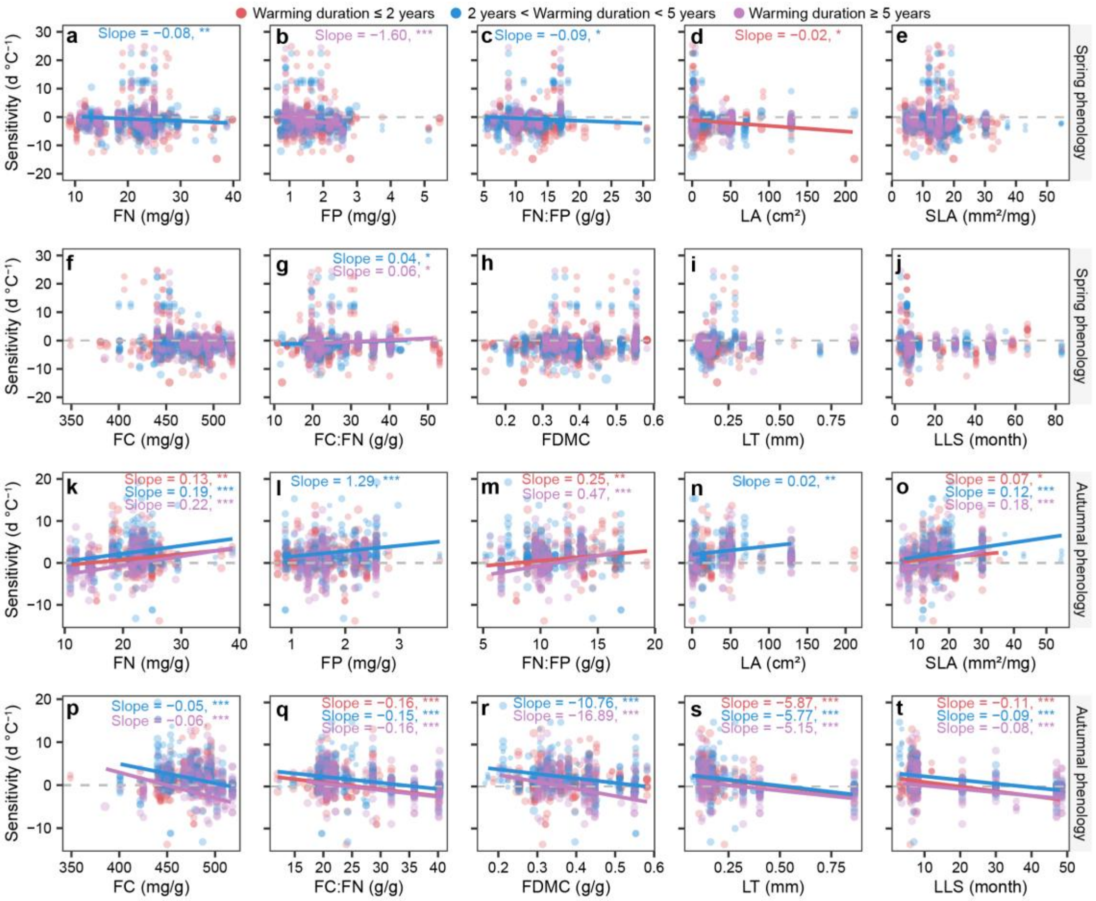

Supplementary Fig.3 Relationships between foliar functional traits and foliar phenology temperature sensitivity based on experimental manipulations under different warming duration. The foliar phenology includes spring phenology (**a**–**j**) and autumnal phenology (**k**–**t**). Statistical significance ($P < 0.05$) was tested using two-sided tests from multi-level meta-analytic linear mixed-effects models without adjustments for multiple comparisons. Regression lines are shown when $P < 0.05$. The size of the points is proportional to the weight in this meta-analysis. FN, foliar nitrogen concentration; FP, foliar phosphorus concentration; FN:FP, foliar nitrogen/phosphorus ratio; LA, leaf area; SLA, specific leaf area; FC: foliar carbon concentration; FC:FN, foliar carbon/nitrogen ratio; FDMC, foliar dry-matter concentration; LT, leaf thickness; LLS, leaf lifespan. *, $P < 0.05$; **, $0.001 < P < 0.01$; ***, $P < 0.001$. Source data are provided as a Source Data file.

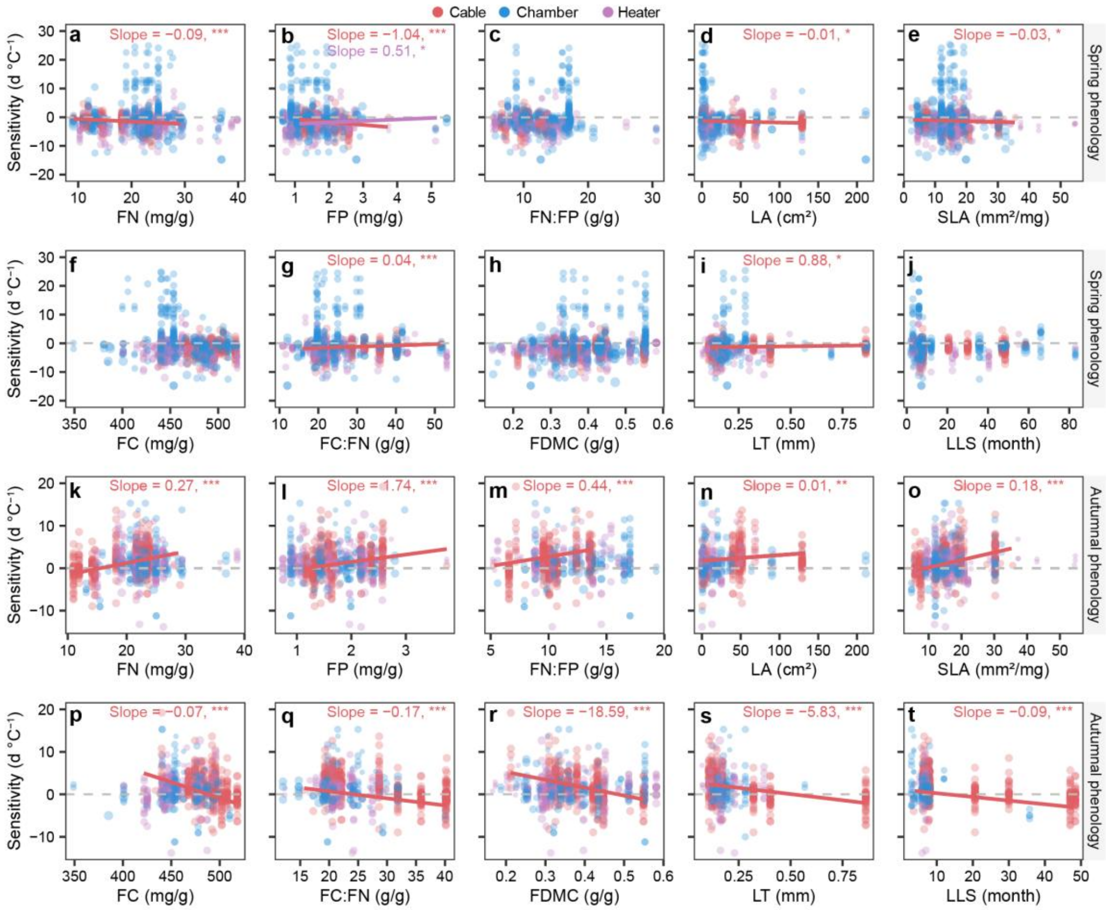

Supplementary Fig.4 Relationships between foliar functional traits and foliar phenology temperature sensitivity based on experimental manipulations under different warming methods. The foliar phenology includes spring phenology (**a**–**j**) and autumnal phenology (**k**–**t**). Statistical significance ($P < 0.05$) was tested using two-sided tests from multi-level meta-analytic linear mixed-effects models without adjustments for multiple comparisons. Regression lines are shown when $P < 0.05$. The size of the points is proportional to the weight in this meta-analysis. FN, foliar nitrogen concentration; FP, foliar phosphorus concentration; FN:FP, foliar nitrogen/phosphorus ratio; LA, leaf area; SLA, specific leaf area; FC: foliar carbon concentration; FC:FN, foliar carbon/nitrogen ratio; FDMC, foliar dry-matter concentration; LT, leaf thickness; LLS, leaf lifespan. *, $P < 0.05$; **, $0.001 < P < 0.01$; ***, $P < 0.001$. Source data are provided as a Source Data file.

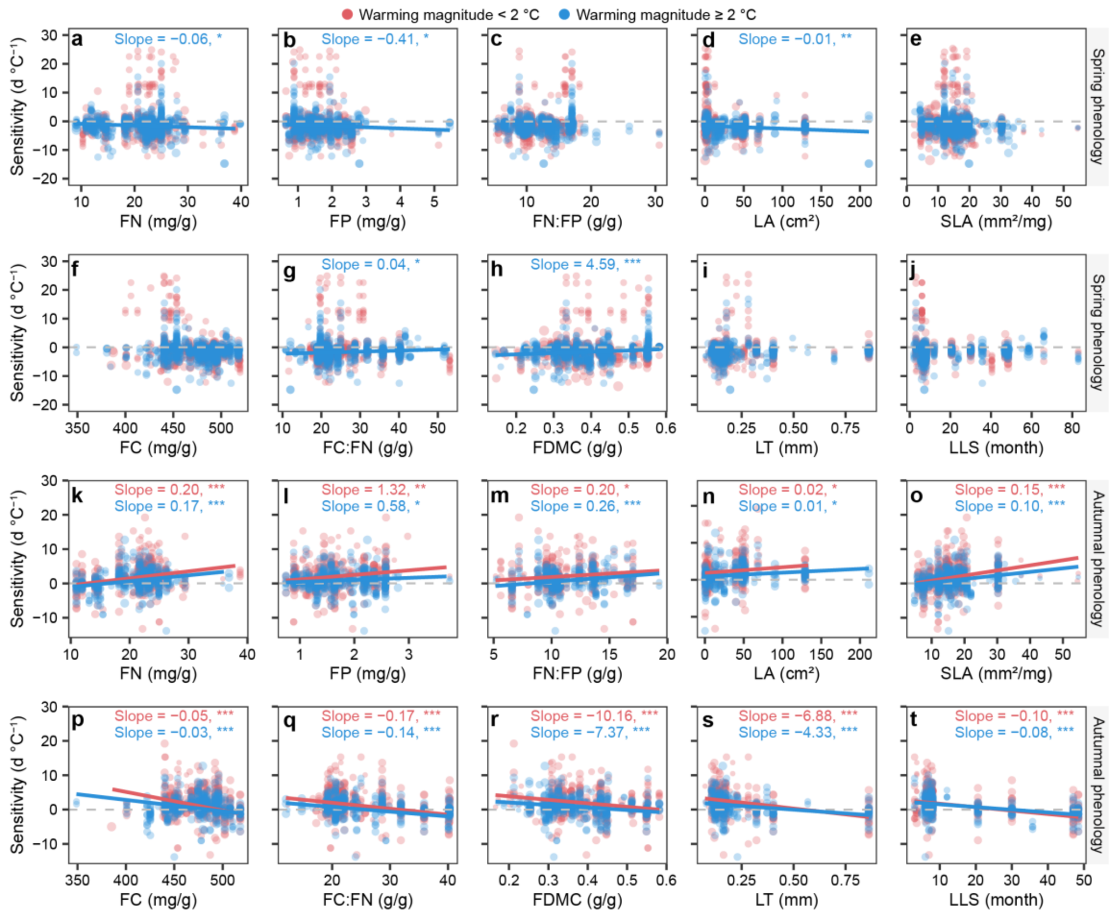

Supplementary Fig.5 Relationships between foliar functional traits and foliar phenology temperature sensitivity based on experimental manipulations under different warming magnitude. The foliar phenology includes spring phenology (**a**–**j**) and autumnal phenology (**k**–**t**). Statistical significance ($P < 0.05$) was tested using two-sided tests from multi-level meta-analytic linear mixed-effects models without adjustments for multiple comparisons. Regression lines are shown when $P < 0.05$. The size of the points is proportional to the weight in this meta-analysis. FN, foliar nitrogen concentration; FP, foliar phosphorus concentration; FN:FP, foliar nitrogen/phosphorus ratio; LA, leaf area; SLA, specific leaf area; FC: foliar carbon concentration; FC:FN, foliar carbon/nitrogen ratio; FDMC, foliar dry-matter concentration; LT, leaf thickness; LLS, leaf lifespan. *, $P < 0.05$; **, $0.001 < P < 0.01$; ***, $P < 0.001$. Source data are provided as a Source Data file.

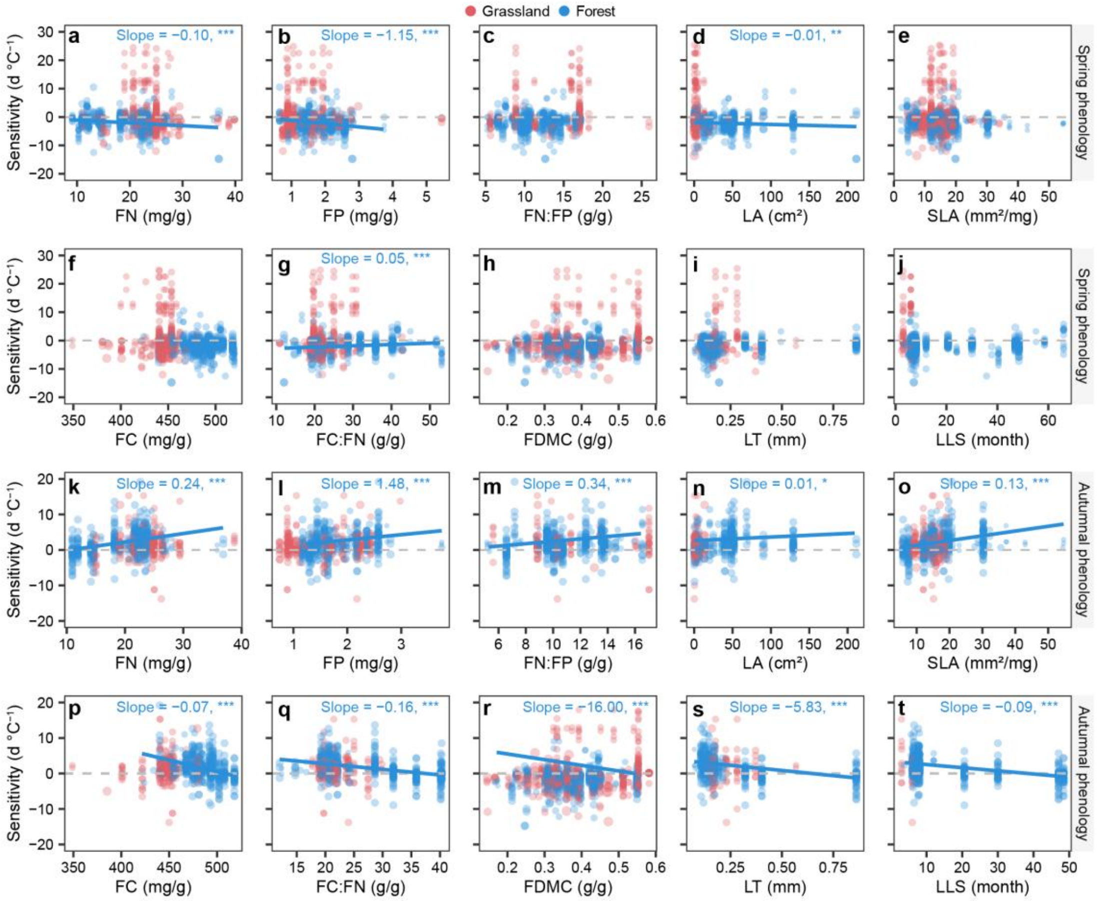

Supplementary Fig.6 Relationships between foliar functional traits and foliar phenology temperature sensitivity based on experimental manipulations under different ecosystem types. The foliar phenology includes spring phenology (**a**–**j**) and autumnal phenology (**k**–**t**). Only field experiments data is included in the analysis. Statistical significance ($P < 0.05$) was tested using two-sided tests from multi-level meta-analytic linear mixed-effects models without adjustments for multiple comparisons. Regression lines are shown when $P < 0.05$. The size of the points is proportional to the weight in this meta-analysis. FN, foliar nitrogen concentration; FP, foliar phosphorus concentration; FN:FP, foliar nitrogen/phosphorus ratio; LA, leaf area; SLA, specific leaf area; FC: foliar carbon concentration; FC:FN, foliar carbon/nitrogen ratio; FDMC, foliar dry-matter concentration; LT, leaf thickness; LLS, leaf lifespan. *, $P < 0.05$; **, $0.001 < P < 0.01$; ***, $P < 0.001$. Source data are provided as a Source Data file.

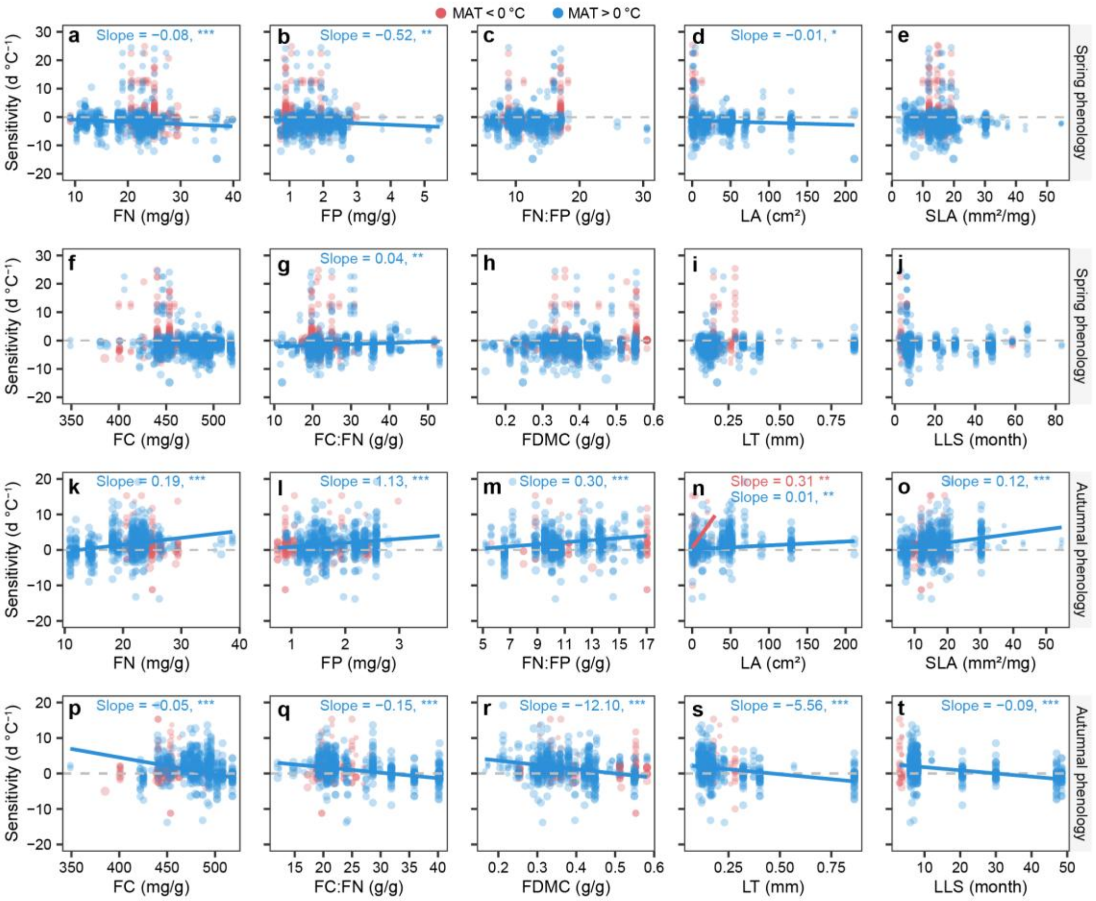

Supplementary Fig.7 Relationships between foliar functional traits and foliar phenology temperature sensitivity based on experimental manipulations under different MAT. The foliar phenology includes spring phenology (**a**–**j**) and autumnal phenology (**k**–**t**). Only field experiments data is included in the analysis. Statistical significance ($P < 0.05$) was tested using two-sided tests from multi-level meta-analytic linear mixed-effects models without adjustments for multiple comparisons. Regression lines are shown when $P < 0.05$. The size of the points is proportional to the weight in this meta-analysis. MAT, mean annual temperature; FN, foliar nitrogen concentration; FP, foliar phosphorus concentration; FN:FP, foliar nitrogen/phosphorus ratio; LA, leaf area; SLA, specific leaf area; FC: foliar carbon concentration; FC:FN, foliar carbon/nitrogen ratio; FDMC, foliar dry-matter concentration; LT, leaf thickness; LLS, leaf lifespan. *, $P < 0.05$; **, $0.001 < P < 0.01$; ***, $P < 0.001$. Source data are provided as a Source Data file.

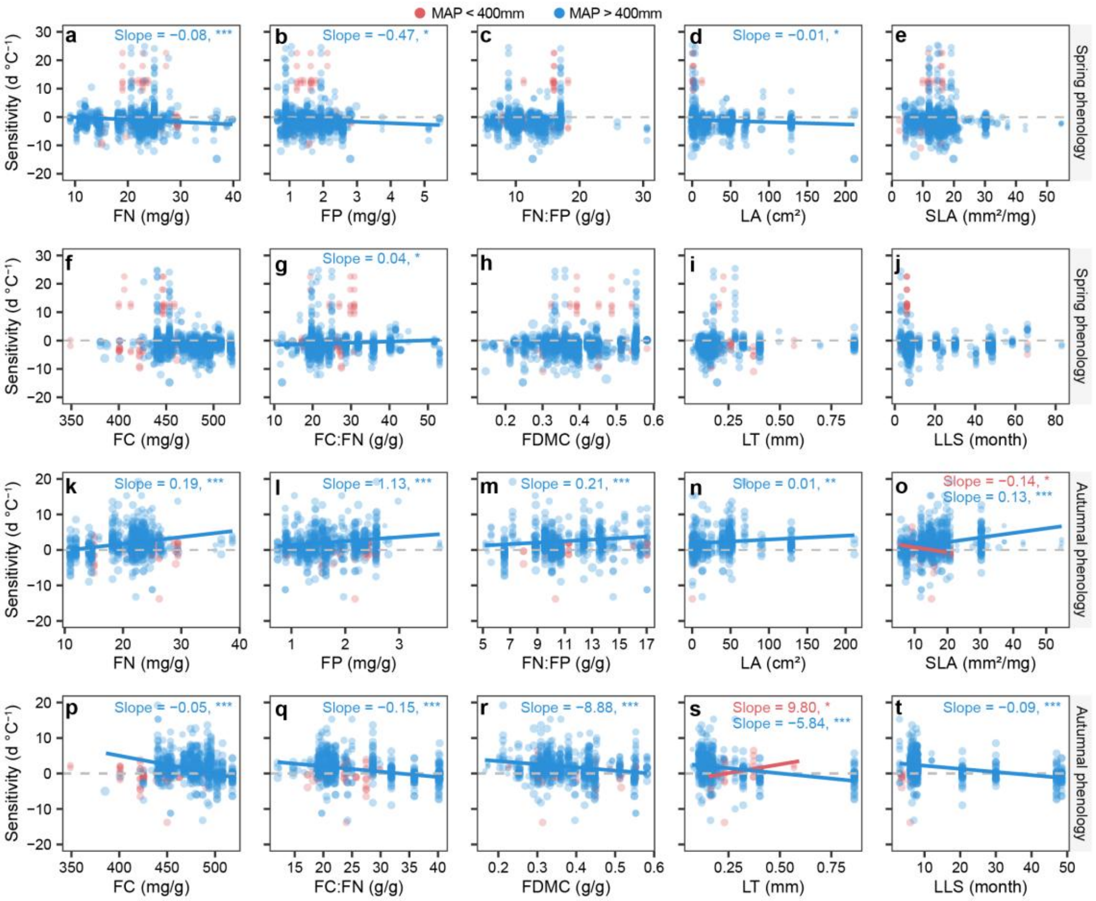

Supplementary Fig.8 Relationships between foliar functional traits and foliar phenology temperature sensitivity based on experimental manipulations under different MAP. The foliar phenology includes spring phenology (**a**–**j**) and autumnal phenology (**k**–**t**). Only field experiments data is included in the analysis. Statistical significance ($P < 0.05$) was tested using two-sided tests from multi-level meta-analytic linear mixed-effects models without adjustments for multiple comparisons. Regression lines are shown when $P < 0.05$. The size of the points is proportional to the weight in this meta-analysis. MAP, mean annual precipitation. FN, foliar nitrogen concentration; FP, foliar phosphorus concentration; FN:FP, foliar nitrogen/phosphorus ratio; LA, leaf area; SLA, specific leaf area; FC: foliar carbon concentration; FC:FN, foliar carbon/nitrogen ratio; FDMC, foliar dry-matter concentration; LT, leaf thickness; LLS, leaf lifespan. *, $P < 0.05$; **, $0.001 < P < 0.01$; ***, $P < 0.001$. Source data are provided as a Source Data file.

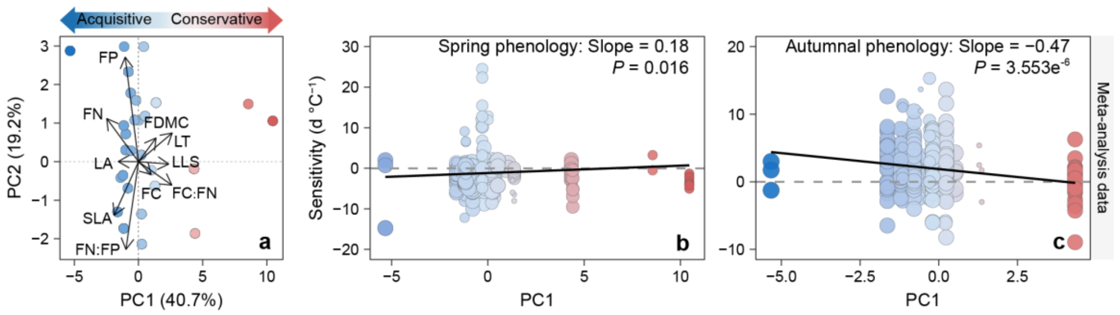

Supplementary Fig.9 Contrasting phenological responses to warming among species with different resource-use strategies based on experimental manipulations. **a**, The leaf economic spectrum based on ten plant traits, where plants with bluer and redder colors are more resource-acquisitive and -conservative, respectively, in their resource use. **b**, **c**, Relationships between the first principal component (PC1) of the economic spectrum and the sensitivities of spring and autumnal phenology to temperature. In (**a**), FN, foliar nitrogen concentration; FP, foliar phosphorus concentration; FN:FP, foliar nitrogen/phosphorus ratio; LA, leaf area; SLA, specific leaf area; FC: foliar carbon concentration; FC:FN, foliar carbon/nitrogen ratio; FDMC, foliar dry-matter concentration; LT, leaf thickness; LLS, leaf lifespan. In (**b** and **c**), statistical significance (P < 0.05) was tested using two-sided tests from multi-level meta-analytic linear mixed-effects models without adjustments for multiple comparisons. Regression lines are shown when P < 0.05. The size of the points is proportional to their weight in this meta-analysis. Source data are provided as a Source Data file.

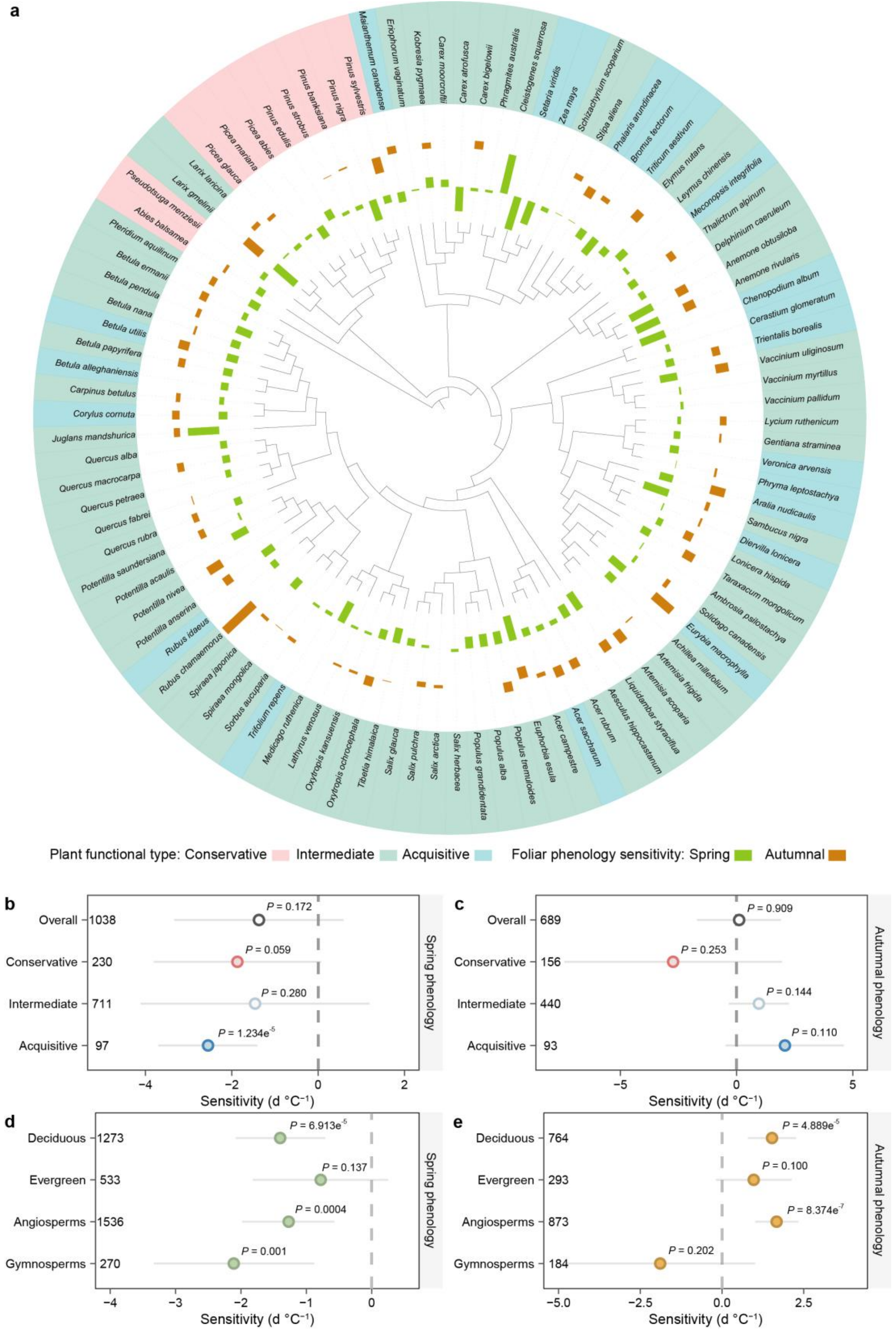

Supplementary Fig.10 Contrasting phenological responses to climate warming among species with different resource-use strategies based on clustering analysis after controlling for phylogenetic relatedness. a, Phylogenetic trees of studied species in meta-analysis and

effect of experimental warming on the temperature sensitivity of their foliar phenology. **b**, **c**, Comparison of the sensitivities of spring and autumnal phenology to temperature across strategy groups after controlling for phylogenetic relatedness. **d**, **e**, Temperature sensitivity of species with different growth habits. In (**a**), The colors of the labels denote resource-use strategy groups. The length of the bar indicates the effect size of warming, with a bar pointing outward (inward) representing a positive (negative) value. In (**b**-**e**), statistical significance ($P < 0.05$) was tested using two-sided tests from multi-level meta-analytic linear mixed-effects models without adjustments for multiple comparisons. Points with error bars represent mean values and 95% confidence intervals (CIs), with vertical dashed lines representing an effect size of zero. Warming effects are significant if the 95% CIs do not overlap with zero. The numbers on the left denote the sample size. Source data are provided as a Source Data file.

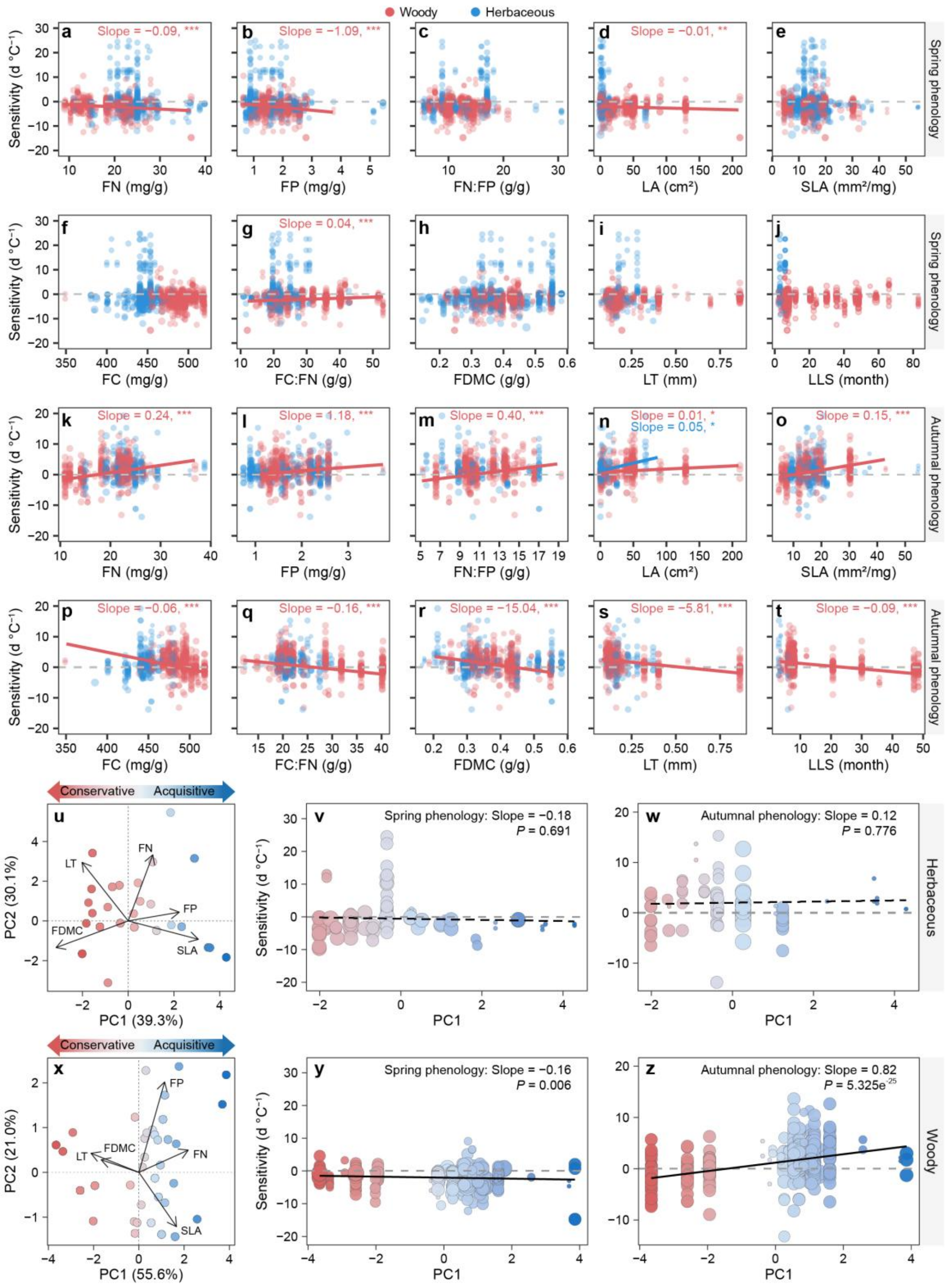

Supplementary Fig.11 Relationships between foliar functional traits and foliar phenology temperature sensitivity based on experimental manipulations under different plant life forms. The foliar phenology includes spring phenology (**a**–**j**) and autumnal phenology (**k**–**t**). Statistical significance (P < 0.05) was tested using two-sided tests from multi-level meta-analytic linear mixed-effects models without adjustments for multiple comparisons. Regression lines are shown when $P < 0.05$. The size of the points is proportional to the weight in this meta-

analysis. **u**, **x**, Leaf economic spectrum derived from functional traits, with color gradients representing resource-use strategies. The red-to-blue continuum indicates resource-conservative (low SLA, high FDMC) to resource-acquisitive (high SLA, low FDMC) species. **v**, **w**, **y** and **z,** Relationships between leaf economic spectrum principal component (PC1) and the sensitivities of spring and autumnal phenology to temperature. Solid regression lines indicate significant correlations (P < 0.05). Dashed regression lines denote nonsignificant correlations (P > 0.05). FN, foliar nitrogen concentration; FP, foliar phosphorus concentration; FN:FP, foliar nitrogen/phosphorus ratio; LA, leaf area; SLA, specific leaf area; FC: foliar carbon concentration; FC:FN, foliar carbon/nitrogen ratio; FDMC, foliar dry-matter concentration; LT, leaf thickness; LLS, leaf lifespan. *, P < 0.05; **, 0.001 < P < 0.01; ***, P < 0.001. Source data are provided as a Source Data file.

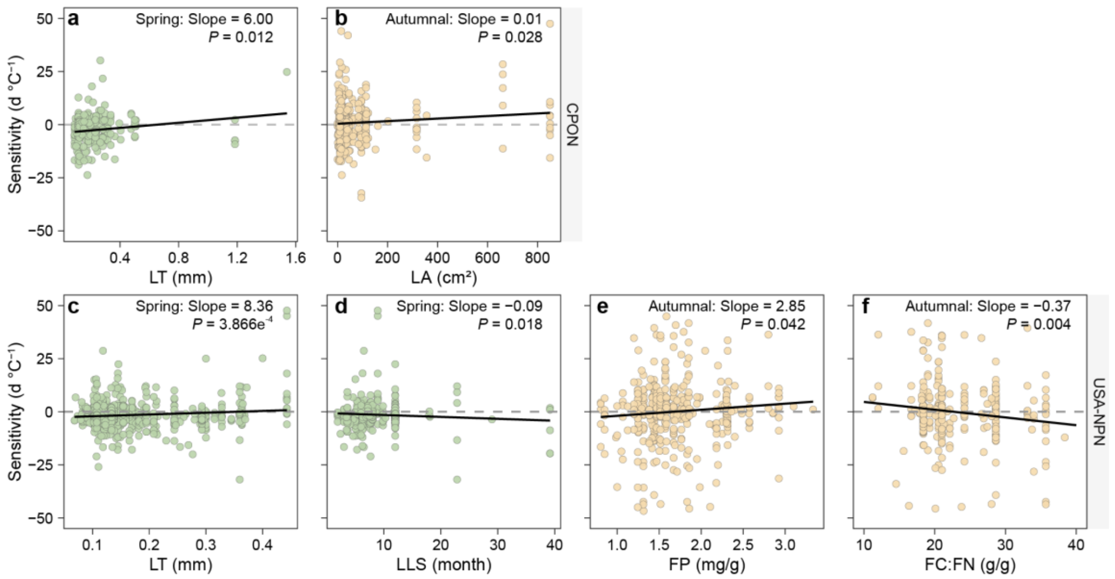

Supplementary Fig.12 Relationships between foliar functional traits and foliar phenology temperature sensitivity based on long-term ground observations. The dataset includes China Phenological Observation Network (CPON) (**a**, **b**) and the USA National Phenology Network (USA-NPN) (**c-f**). Statistical significance ($P < 0.05$) was tested using two-sided tests from multi-level meta-analytic linear mixed-effects models without adjustments for multiple comparisons. Regression lines are shown when $P < 0.05$. LT, leaf thickness; LA, leaf area; LLS, leaf lifespan; FP, foliar phosphorus concentration; FC:FN, foliar carbon/nitrogen ratio. Source data are provided as a Source Data file.

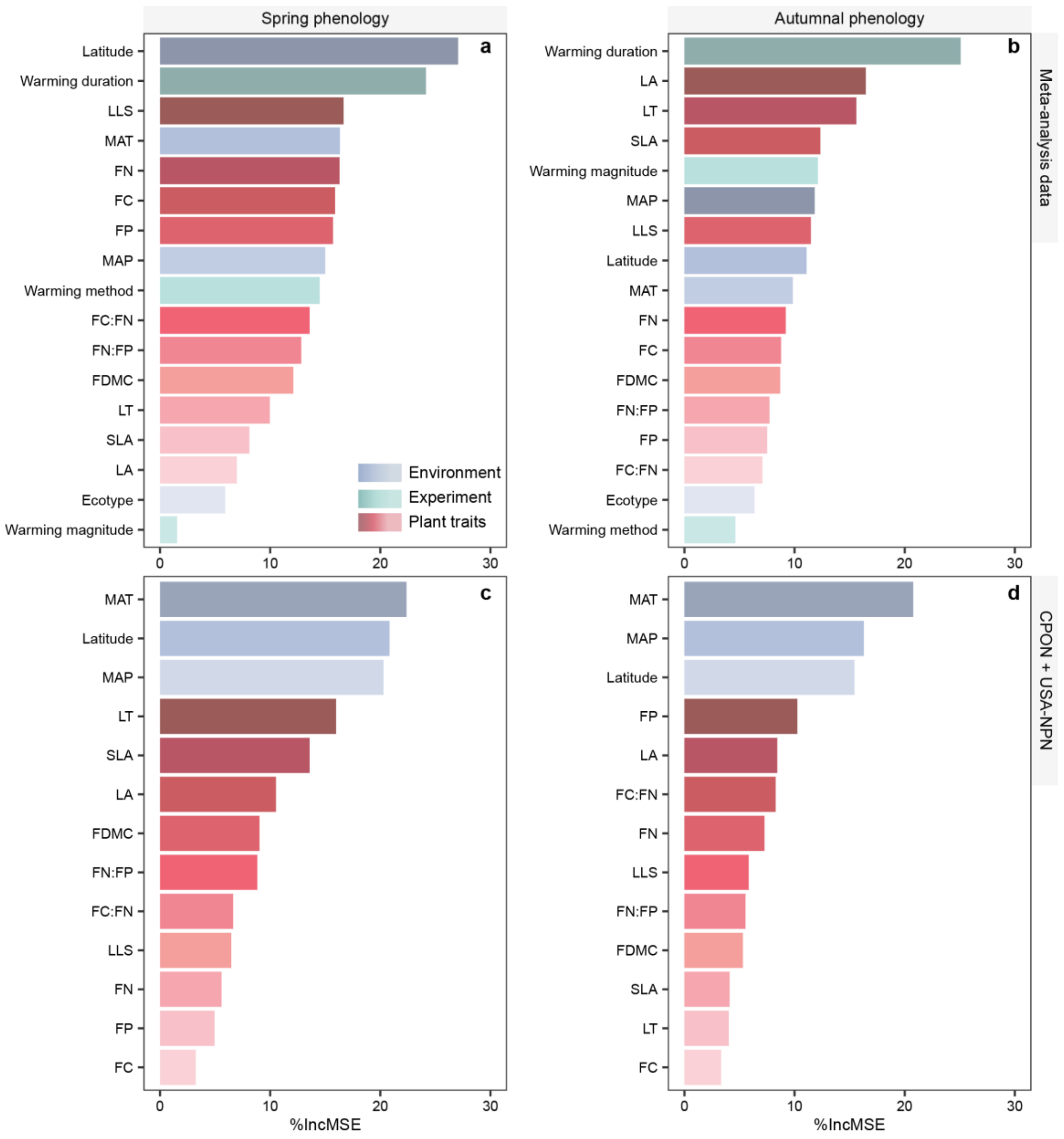

Supplementary Fig.13 Ranking of key drivers influencing foliar phenological temperature sensitivity using %IncMSE based on random forest analysis. a, **b**, Ranking of factors influencing spring and autumnal phenological sensitivity in warming experiments. For meta-analysis data, only field experiments data is included in the analysis. **c**, **d**, Ranking of factors influencing phenological sensitivity based on two long-term ground phenological datasets. FN, foliar nitrogen concentration; FP, foliar phosphorus concentration; FN:FP, foliar nitrogen/phosphorus ratio; LA, leaf area; SLA, specific leaf area; FC: foliar carbon concentration; FC:FN, foliar carbon/nitrogen ratio; FDMC, foliar dry-matter concentration; LT, leaf thickness; LLS, leaf lifespan; MAT, mean annual temperature; MAP, mean annual precipitation; Ecotype, ecosystem type. Source data are provided as a Source Data file.

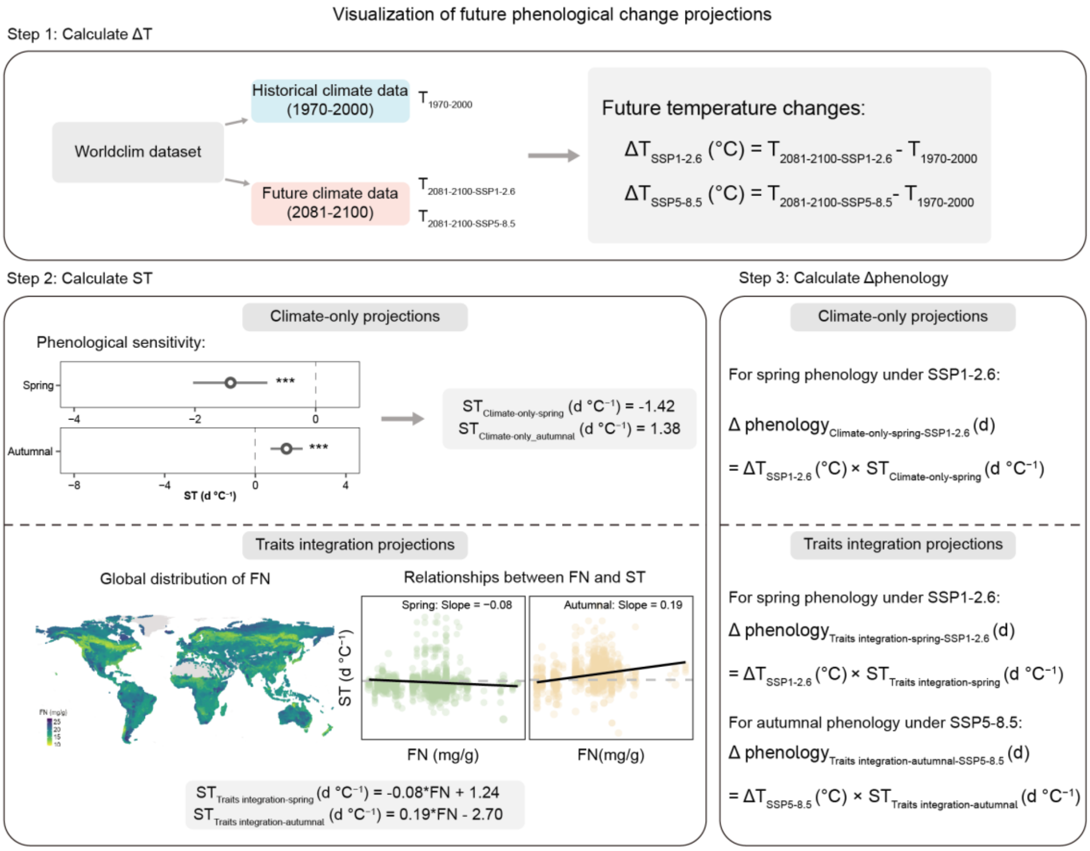

Supplementary Fig.14 Visualization of future phenological change projections. SSP, Shared Socioeconomic Paths. ΔT, The changes of temperature. SSP, Shared Socioeconomic Paths. ST, phenological temperature sensitivity. FN, foliar nitrogen concentration. Δ phenology, shifts in foliar phenology.

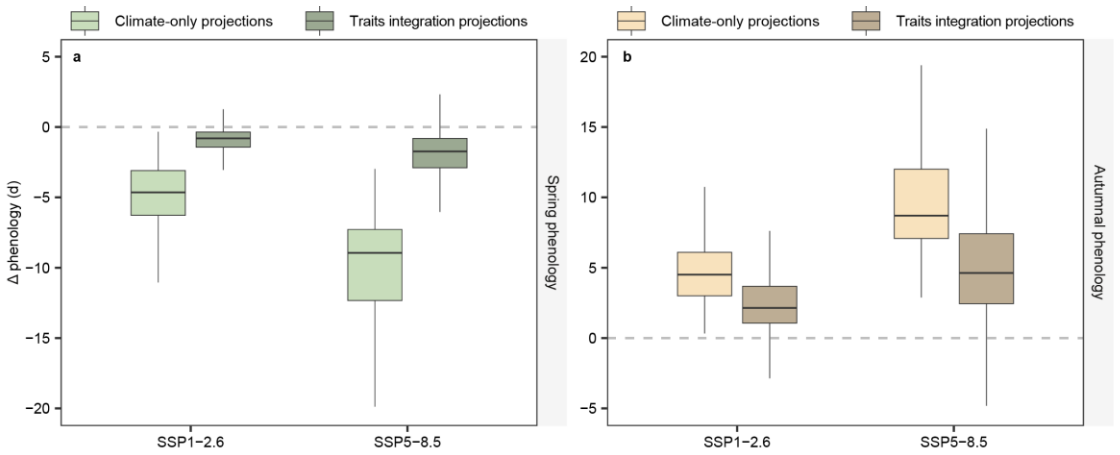

Supplementary Fig.15 Future spring and autumnal phenological changes based on climate-only and traits integration projections by 2100 under two climatic scenarios. SSP, Shared Socioeconomic Paths. Δ phenology, shifts in foliar phenology. Source data are provided as a Source Data file.

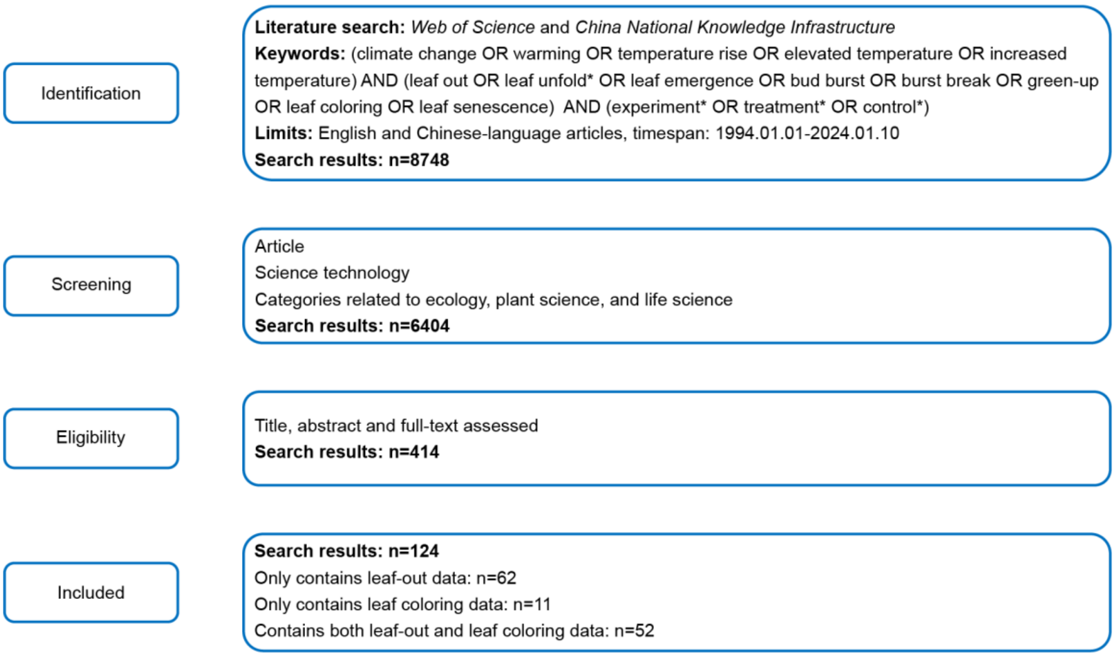

Supplementary Fig.16 Processes of article selection for effects of warming on plant foliar phenology using Preferred Reporting Items for Systematic Reviews (PRISMA) guidelines.

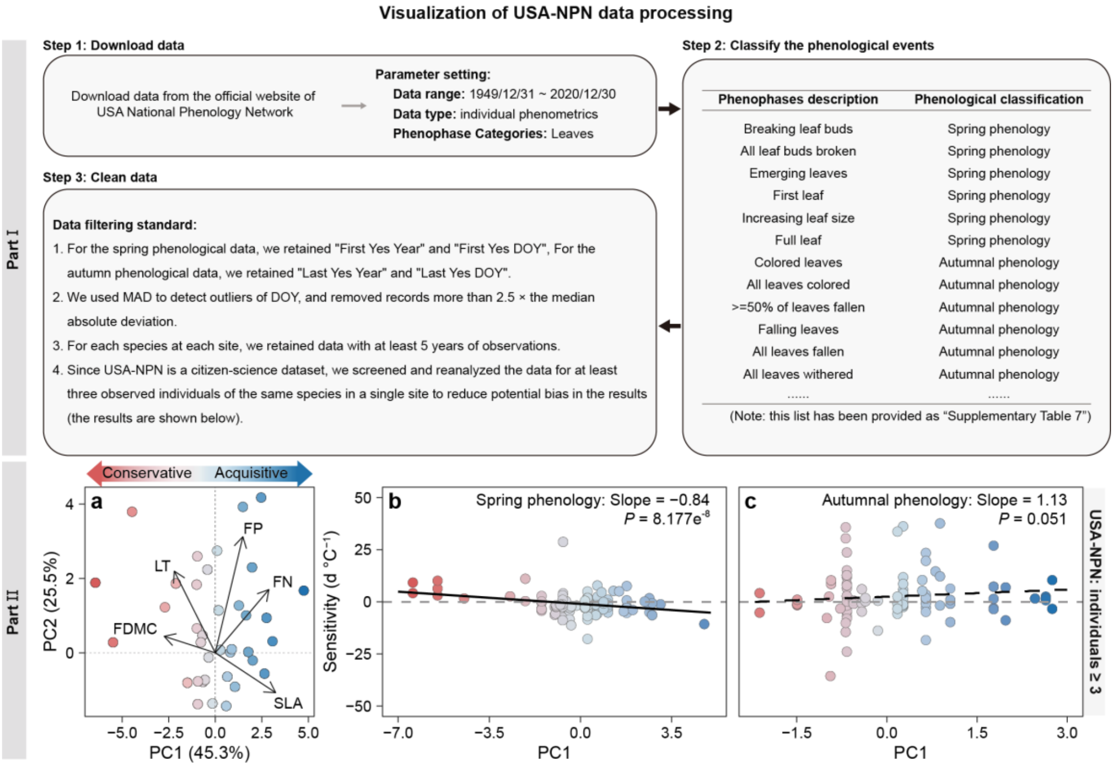

Supplementary Fig.17 Visualization of USA National Phenology Network data processing. In part I, First Yes Year, the year of the first "yes" record of the series; First Yes DOY, the day of year, ranging from 1 to 366, of the first "yes" record of the series; Last Yes Year, the year of the last "yes" record of the series; Last Yes DOY, the day of year, ranging from 1 to 366, of the last "yes" record of the series; MAD, median absolute deviation; DOY, day of year. In part II, we screened and reanalyzed the data of at least three observed individuals of the same species in a single site to enhance the robustness of the results. **a**, The leaf economic spectrum based on five plant traits, where plants with redder and bluer colors are more resource-conservative and -acquisitive, respectively, in their resource use. **b**, **c**, Relationships between the first principal component (PC1) of the economic spectrum and the sensitivities of spring and autumnal phenology to temperature. Statistical significance ($P < 0.05$) was tested using two-sided tests from multi-level meta-analytic linear mixed-effects models without adjustments for multiple comparisons. Solid regression lines indicate significant correlations ($P < 0.05$). Dashed regression lines denote nonsignificant correlations ($P > 0.05$). In (**a**), FN, foliar nitrogen concentration; FP, foliar phosphorus concentration; SLA, specific leaf area; FDMC, foliar dry-matter concentration; LT, leaf thickness. Source data are provided as a Source Data file.
