# Supplementary material for: Acquisitive plants exhibit stronger phenological shifts in response to warming: insights from meta-analysis and long-term monitoring
Source: Nat Commun. 2026 Mar 26;17:4430. doi: 10.1038/s41467-026-70474-4 (PMC13183894; doi:10.1038/s41467-026-70474-4)
Supplement: Supplementary file 2 — Reporting Summary [file 41467_2026_70474_MOESM2_ESM.pdf]

nature portfolio

Corresponding author(s): Chaoyang Wu
Huiying Liu

Last updated by author(s): Feb 21, 2026

Reporting Summary

Nature Portfolio wishes to improve the reproducibility of the work that we publish. This form provides structure for consistency and transparency in reporting. For further information on Nature Portfolio policies, see our Editorial Policies and the Editorial Policy Checklist.

Statistics

For all statistical analyses, confirm that the following items are present in the figure legend, table legend, main text, or Methods section.

n/a	Confirmed	
☐	☒	The exact sample size (*n*) for each experimental group/condition, given as a discrete number and unit of measurement
☐	☒	A statement on whether measurements were taken from distinct samples or whether the same sample was measured repeatedly
☐	☒	The statistical test(s) used AND whether they are one- or two-sided<br>*Only common tests should be described solely by name; describe more complex techniques in the Methods section.*
☐	☒	A description of all covariates tested
☐	☒	A description of any assumptions or corrections, such as tests of normality and adjustment for multiple comparisons
☐	☒	A full description of the statistical parameters including central tendency (e.g. means) or other basic estimates (e.g. regression coefficient) AND variation (e.g. standard deviation) or associated estimates of uncertainty (e.g. confidence intervals)
☐	☒	For null hypothesis testing, the test statistic (e.g. *F*, *t*, *r*) with confidence intervals, effect sizes, degrees of freedom and *P* value noted<br>*Give P values as exact values whenever suitable.*
☒	☐	For Bayesian analysis, information on the choice of priors and Markov chain Monte Carlo settings
☒	☐	For hierarchical and complex designs, identification of the appropriate level for tests and full reporting of outcomes
☒	☐	Estimates of effect sizes (e.g. Cohen's *d*, Pearson's *r*), indicating how they were calculated

Our web collection on statistics for biologists contains articles on many of the points above.

Software and code

Policy information about availability of computer code

Data collection	In the meta-analysis section, if the phenological data cannot be directly extracted from the tables and appendices, we use WebPlotDigitizer (version 4.7) to extract them from the graphical figures and plots.
Data analysis	All analyses were conducted in the R software (version4.3.2) and all analysis codes are publicly available through open-source R package, including 'metafor' package (version 4.4-0), 'V.phyloMaker' package (version 0.1.0), 'ape' package (version 5.8-1), 'picante' package (version 1.8.2), 'stats' package (version 4.3.2), 'FactoMineR' (version 2.9), 'Hmisc' package (version 5.2-2), 'nlme' package (version 3.1-163) and 'randomForest' package (version 4.7-1.2).

For manuscripts utilizing custom algorithms or software that are central to the research but not yet described in published literature, software must be made available to editors and reviewers. We strongly encourage code deposition in a community repository (e.g. GitHub). See the Nature Portfolio guidelines for submitting code & software for further information.

Data

Policy information about availability of data

All manuscripts must include a data availability statement. This statement should provide the following information, where applicable:
- Accession codes, unique identifiers, or web links for publicly available datasets
- A description of any restrictions on data availability
- For clinical datasets or third party data, please ensure that the statement adheres to our policy

The data generated in this study have been deposited in Figshare at https://doi.org/10.6084/m9.figshare.29917052. Plant traits data were obtained from TRY Plant

Trait Database (https://www.try-db.org/), climate data from WorldClim (https://www.worldclim.org/) and Climate Research Unit (https://crudata.uea.ac.uk/cru/data/hrg/cru_ts_4.08/). The long-term ground phenological data of USA National Phenology Network (USA-NPN) are available from the website: https://www.usanpn.org/ results/data. The phenological data from China Phenological Observation Network (CPON) were provided by the Meteorological Information Center of the China Meteorological Administration. Source data are provided with this paper.

Research involving human participants, their data, or biological material

Policy information about studies with human participants or human data. See also policy information about sex, gender (identity/presentation), and sexual orientation and race, ethnicity and racism.

Reporting on sex and gender	N/A
Reporting on race, ethnicity, or other socially relevant groupings	N/A
Population characteristics	N/A
Recruitment	N/A
Ethics oversight	N/A

Note that full information on the approval of the study protocol must also be provided in the manuscript.

Field-specific reporting

Please select the one below that is the best fit for your research. If you are not sure, read the appropriate sections before making your selection.

☐ Life sciences ☐ Behavioural & social sciences ☒ Ecological, evolutionary & environmental sciences

For a reference copy of the document with all sections, see nature.com/documents/nr-reporting-summary-flat.pdf

Ecological, evolutionary & environmental sciences study design

All studies must disclose on these points even when the disclosure is negative.

Study description	We combine a meta-analysis of 124 field warming experiments with data from two long-term phenological observation networks (USA-NPN, CPON) to examine the influence of plant functional traits on phenological shifts.
Research sample	In the meta-analysis section, our screening process produced 3079 observations from 124 peer-reviewed articles published, comprising 1941 observations for spring phenology and 1138 observations for autumnal phenology.<br>In the section of long-term ground phenological observations data analysis, we constructed the database for the long-term ground monitoring of spring and autumnal phenology using data from the China Phenology Observation Network (CPON, 1982-2018; http://www.cpon.ac.cn) and the USA National Phenology Network (USA-NPN, 1949-2020; https://www.usanpn.org/ results/data). The final database encompassed 395 observation sites (30-55°N) and 705 taxa of woody plants (spanning 114 families and 339 genera), comprising 40098 observations of foliar phenological sequences.
Sampling strategy	As for meta-analysis data, we then included studies that met the following criteria: (i) warming experiments were conducted in terrestrial ecosystems, (ii) initial environmental conditions were comparable between control and warming plots, (iii) the method, duration, and magnitude of warming were clearly described, (iv) experimental species were known and indicated, and (v) the timing of phenological events (measured as day of year) under both warming and control treatments, or the phenological shifts induced by warming (in days), along with their sample sizes, were reported.<br>As for long-term ground phenological observations data, we applied temporal continuity thresholds (CPON ≥10 y, USA-NPN ≥5 y) to filter valid observational records. Robust statistical methods were used for detecting outliers, with records more than 2.5× the median absolute deviation excluded. Since USA-NPN is a citizen-science dataset, we screened and reanalyzed the data of at least three observed individuals of the same species in a single site to reduce potential bias in the results.
Data collection	Meta-analysis data were collected by KX, CL and HZ. Specifically, relevant peer-reviewed literature was identified through systematic searches of Web of Science, Google Scholar, and the China National Knowledge Infrastructure, using the following key words: (climate change OR warming OR temperature rise OR elevated temperature OR increased temperature) AND (leaf out OR leaf unfold* OR leaf emergence OR bud burst OR burst break OR green-up OR leaf color OR leaf senescence) AND (experiment* OR treatment* OR control*).<br>Long-term ground phenological observations data were collected by KX from the China Phenology Observation Network (CPON, http:// www.cpon.ac.cn) and the USA National Phenology Network (USA-NPN, https://www.usanpn.org/ results/data).
Timing and spatial scale	As for meta-analysis data, we collected all relevant articles published before January 2024 on a global scale.<br>As for long-term ground phenological observations data, we collected the data of China Phenology Observation Network from 1982 to 2018 and that of the USA National Phenology Network from 1949 to 2020.

Data exclusions	In the meta-analysis section, studies were excluded if they did not meet the data selection criteria reported above.<br>In the section of long-term ground phenological observations data analysis, median absolute deviation was used to exclude outliers from the CPON and USA-NPN datasets. In addition, for the CPON dataset, we retained phenological observations data for more than 10 years, and for USA-NPN, we retained phenological observations data for more than 5 years.
Reproducibility	The findings of our study can be reproduced following the methods of data collection and analysis presented in the Methods section in the manuscript.
Randomization	Randomization is not applicable to a meta-analysis. And because the long-term ground phenological observations data in our study were obtained from open-access database instead of designed experiments, randomization is also not applicable.
Blinding	Blinding is not applicable to a meta-analysis. And because the long-term ground phenological observations data in our study were obtained from open-access database instead of designed experiments, blinding is also not applicable.

Did the study involve field work? ☐ Yes ☒ No

Reporting for specific materials, systems and methods

We require information from authors about some types of materials, experimental systems and methods used in many studies. Here, indicate whether each material, system or method listed is relevant to your study. If you are not sure if a list item applies to your research, read the appropriate section before selecting a response.

Materials & experimental systems

n/a	Involved in the study	
☒	☐	Antibodies
☒	☐	Eukaryotic cell lines
☒	☐	Palaeontology and archaeology
☒	☐	Animals and other organisms
☒	☐	Clinical data
☒	☐	Dual use research of concern
☒	☐	Plants

Methods

n/a	Involved in the study	
☒	☐	ChIP-seq
☒	☐	Flow cytometry
☒	☐	MRI-based neuroimaging

Plants

Seed stocks	N/A
Novel plant genotypes	N/A
Authentication	N/A
